# Monstra Abundantia

**Published:** 1868-03

**Authors:** A. H. De Puy

**Affiliations:** Toronto, Ind.


### MONSTRA ABUNDANTIA.—A CASE OF DIFFICULT LABOR FROM DEFORMITY OF THE FŒTUS.

BY A. H. DE PUY, M.D., Toronto, Ind.

Being called to an obstetrical case, a short time ago, in which the diagnosis was very obscure, and the case rendered tedious and protracted in consequence of the deformed state of the child, which proved to be abdominal ascites. It contained about 11 pints of fluid, and measured two feet and one inch in circumference. There was nothing unusual in the first stage of labor, nor until the child's head presented at the inferior strait, when it became apparent that nature was not sufficient to terminate the case. I therefore sent for my obstetrical instruments, and my student, Dr. William H. Burtnett also sent a messenger for Dr. Daniel Camerie; all arrived in due time.

The patient being much exhausted, having been in labor 24

hours, we decided to give her an anodyne, and let her rest a short time. After examination and consultation, the pains returning, we succeeded in bringing down the head and arms, using traction; but making no further progress, we concluded next to dissect the head and shoulders, and perform version. This being done, I succeeded in securing the feet and bringing them down. We then introduced the blunt hook over the pubic bones of the child, using considerable force, but of no avail. Then we determined to remove the bowels and lungs, and thus cause a collapse. But, alas! to our surprise, the moment I perforated the abdominal cavity, an enormous quantity of fluid escaped, and the labor was terminated in a few minutes, *per vias naturalis.*

The woman made a good recovery, and is now living in Edgar Co., Ill.; is about 35 years of age, and mother of three children.

